# Assessment of three essential tremor genetic loci in sporadic Parkinson's disease in Eastern China

**DOI:** 10.1111/cns.13272

**Published:** 2019-11-21

**Authors:** Ting Gao, Jiong Wu, Ran Zheng, Yi Fang, Chong‐Yao Jin, Yang Ruan, Jin Cao, Jun Tian, Jia‐Li Pu, Bao‐Rong Zhang

**Affiliations:** ^1^ Department of Neurology College of Medicine Second Affiliated Hospital Zhejiang University Hangzhou China

**Keywords:** *LINGO1*, Parkinson's disease, *PPARGC1A*, single nucleotide polymorphism, *SLC1A2*

## Abstract

**Objective:**

The aim of this study was to investigate potential genetic overlap between essential tremor and Parkinson's disease in a cohort of 825 subjects from an Eastern Chinese population.

**Methods:**

A total of 441 Parkinson's disease patients and 384 healthy controls were recruited. The MassARRAY System was used to detect three essential tremor‐related single nucleotide polymorphisms. Odds ratio (OR) and 95% confidential interval (CI) were calculated to assess the relationship between polymorphisms and Parkinson's disease susceptibility.

**Results:**

Our results demonstrated that the odds ratios of rs3794087 of SLC1A2, rs9652490 of LINGO1, and rs17590046 of PPARGC1A were 0.71 (95% CI = 0.55‐0.91), 0.99 (95% CI = 0.78‐1.26), and 0.88 (95% CI = 0.62‐1.25), respectively.

**Conclusion:**

An essential tremor SNP (rs3794087 of SLC1A2) is associated with a decreased risk of PD in the Eastern Han Chinese population, while rs9652490 (*LINGO1*) and rs17590046 (*PPARGC1A*) do not show an association.

## 1 INTRODUCTION

Parkinson's disease (PD) and essential tremor (ET) are both common neurodegenerative disorders, whose prevalence increases with age. PD affects 1.8% of people over the age of 65^1^ and is characterized by resting tremor, rigidity, bradykinesia, and gait disturbances. ET affects greater than 5% of the population over 65 years of age^2^ and is characterized mainly by action tremor involving the upper extremities. The tremor can be postural or kinetic in association with voluntary muscle contraction. While the majority of PD cases (90%) are sporadic,^3^ ET is commonly familial (50%‐70% of cases)^4^ and tends to start earlier in the age, following a more benign clinical course. Whereas PD and ET are generally regarded as distinct entities, both conditions share some motor and nonmotor clinical features such as resting tremor, postural instability, olfactory deficits, cognitive disturbances, and increased frequency of rapid eye movement behavior disorder (RBD).^5^ Additionally, Spanaki et al^6^ showed that ET appeared more frequently in relatives of PD patients than in controls (odds ratio (OR): 3.64, *P* < .001) After the initial diagnosis of ET, the risk of developing a typical PD is 4‐fold greater than that of the non‐ET population.^1^ The overlapping clinical features and epidemiological findings suggest that PD and ET may share common genetic risk factors. In order to address the genetic relationship between ET and PD, we conducted a case‐control association study of three variants (LINGO1 rs9652490; SLC1A2 rs3794087; and PPARGC1A rs17590046) that were previously reported to be associated with ET. Among them, LINGO1 rs9652490 and SLC1A2 rs3794087 were two lead single nucleotide polymorphisms of previous small genome‐wide association studies in ET.^7^, ^8^, ^9^ PPARGC1A rs17590046 was also proved to be associated with essential tremor recently.^10^ But they showed controversial associations in PD studies.

## 2 MATERIALS AND METHODS

### 2.1 Study participants

Parkinson's disease patients were recruited and diagnosed by two movement disorder specialists from outpatient clinics according to the diagnostic criteria provided by the Movement Disorder Society.^11^ Patients with secondary causes of parkinsonism such as vascular, drug‐induced, and toxin‐induced, and other neurodegenerative diseases such as progressive supranuclear palsy, multiple system atrophy, essential tremor, and Wilson's disease were excluded. Unrelated healthy control subjects were enrolled from local communities and evaluated by two movement disorder specialists for exclusion of PD. Written informed consents were signed by all participants. This study was reviewed and approved by the Medical Ethics Committee of the Second Affiliated Hospital of Zhejiang University School of Medicine in accordance with the Declaration of Helsinki.

### 2.2 DNA preparation and genotyping

Two milliliter blood samples were collected from all subjects. DNA was extracted by a plant DNA rapid extraction kit (BioTeKe Corporation). Genotyping assays were designed using AssayDesigner 3.1. The following three single nucleotide polymorphisms (SNPs) were tested: rs3794087 of *SLC1A2*, rs9652490 of *LINGO1*, and rs17590046 of *PPARGC1A*. Agena MassARRAY Typer 4.0 (Agena, Inc) was used to determine the genotypes of these three SNPs in all subjects.

### 2.3 Statistical analysis

Student's *t* test was used to assess differences in age between PD patients and controls. Chi‐square test was used to detect the HWE (Hardy‐Weinberg equilibrium), gender, and allele distribution differences between the two groups. Pearson chi‐square test or Fisher's exact test was performed in risk analysis, and an OR with a 95% confidence interval (95% CI) for each SNP was calculated according to dominant and recessive models. Cochran‐Armitage trend test was conducted to evaluate the OR of the additive model. The strength of polymorphisms and PD susceptibility was assessed by OR. The genetic power calculations were performed in Quanto.^12^ All other statistical analyses were performed in IBM SPSS Statistics 23.0. A two‐tailed *P* < .05 was considered significant for all analyses.

## 3 RESULTS

A total of 441 patients with PD (male/female: 235/206; age: 60.5 ± 11.1 years) and 384 unrelated healthy control participants (male/female: 197/187; age: 59.8 ± 8.3 years) who were natives of east China were included in this study. There were no significant differences in gender or age distribution between patients and controls (Table 1). High genotyping qualities were acquired in all genotyping reactions. All control genotype frequencies in all of the studied polymorphisms were in HWE. In genotypic analysis, the OR of dominant and recessive models of the SNP rs3794087 (*SLC1A2*) was 0.70 (95% CI = 0.51‐0.95, *P* = .023) and 0.47 (95% CI = 0.24‐0.93, *P* = .028), respectively. And the powers of dominant and recessive models were more than 0.6 (Table 2 and Table 3). In allelic analysis, the OR of T allele of rs3794087 was 0.71 (95%CI = 0.55‐0.91, *P* = .007). No significant difference was found in SNPs of LINGO1 and PPARGC1A. The OR of rs9652490 of LINGO1 and rs17590046 of PPARGC1A was 0.99 (95% CI = 0.78‐1.26, *P* = .932) and 0.88 (95% CI = 0.62‐1.25, *P* = .472), respectively (Table 2). Moreover, no discrepancy was detected in MAF (minor allele frequency) between our population and 1000 Genomes in PubMed (Table 4).

**Table 1:** Demographic characteristics

	Control (n = 384)	PD (n = 441)	*P*
Sex (male/female)	197/187	235/206	.569
Age (y), mean ± SD	59.8 ± 8.3	60.5 ± 11.1	.503

Abbreviation: PD, Parkinson's disease.

**Table 2:** Genotypic analysis of three loci, previously established as risk alleles for essential tremor, in Parkinson's disease

SNP (Candidate gene)	HWE	Genotype/Allele	Association test	Control	PD	*P*	OR (95% CI)
**rs3794087 (*SLC1A2*)**	0.814	Genotype	Genotypic (TT/GT/GG)	20/106/151	15/142/269		
			Additional			**.007**	‐
			Dominant ((TT + GT)/GG)	126/151	157/269	**.023**	**0.70 (0.51‐0.95)**
			Recessive (TT/(GT + GG))	20/257	15/411	**.028**	**0.47 (0.24‐0.93)**
		Allele	T/G	146/408	172/680	**.007**	**0.71 (0.55‐0.91)**
rs9652490 (*LINGO1*)	0.072	Genotype	Genotypic (GG/AG/AA)	12/138/216	19/153/263		
			Additional			.930	‐
			Dominant ((GG + AG)/AA)	150/216	172/263	.678	0.94 (0.71‐1.25)
			Recessive (GG/(AG + AA))	12/354	19/416	.426	1.35 (0.65‐2.81)
		Allele	G/A	162/570	191/679	.932	0.99 (0.78‐1.26)
rs17590046 (*PPARGC1A*)	0.967	Genotype	Genotypic (CC/CT/TT)	3/61/302	5/60/365		
			Additional			.480	‐
			Dominant ((CC + CT)/TT)	64/302	65/365	.366	0.84 (0.58‐1.23)
			Recessive (CC/(CT_TT))	3/363	5/425	.899	1.42 (0.34‐6.00)
		Allele	C/T	67/665	70/790	.472	0.88 (0.62‐1.25)

Note

The positive locus identified in this study is marked in bold font.

Abbreviations: CI, confidence interval; HWE, Hardy‐Weinberg equilibrium of control group; OR, odds ratio; PD, Parkinson's disease; SNP, single nucleotide polymorphism.

**Table 3:** Power calculation of three loci (dominant & recessive models)

SNP	model	observed MAF	control	PD	N	CON per case	OR	Power
**rs3794087**	Dominant	0.226	277	426	703	0.65	**0.70**	**0.82**
	Recessive						**0.47**	**0.66**
rs9652490	Dominant	0.220	366	435	801	0.84	0.94	0.09
	Recessive						1.35	0.26
rs17590046	Dominant	0.086	366	430	796	0.85	0.84	0.22
	Recessive						1.42	0.10

Note

The positive locus identified in this study was marked in bold font.

Abbreviations: CON, controls; MAF, minor allele frequency; OR, odds ratio. PD, Parkinson's disease; SNP, single nucleotide polymorphism.

**Table 4:** MAF calculation and comparison

	observed	1000‐G	1000‐GEA
rs3794087	T = 0.226	T = 0.160	T = 0.189
rs9652490	G = 0.220	G = 0.269	G = 0.227
rs17590046	C = 0.086	C = 0.155	C = 0.097

Note

All data from PubMed were collected on August 1 2019.

Abbreviations: 1000‐G, MAF in 1000 Genomes in PubMed; 1000‐GEA, MAF in 1000 Genomes of East Asian populations in PubMed; MAF, minor allele frequency; Observed, MAF in our study.

## 4 DISCUSSION

The association between essential tremor and Parkinson's disease has been a subject of long‐standing debates.^13^, ^14^, ^15^ A study by Jankovic and colleagues was the first to suggest that some patients with essential tremor have a genetically increased risk for PD.^13^ Later, Koller et al^14^ argued that frequency of a family history of ET was higher among PD patients than control subjects but lacked statistically significant difference. Rajput et al^16^ studied nine patients with essential tremor who had autopsies and found the risk of idiopathic Parkinson's disease in essential tremor cases is similar to the general population. Until nowadays, there is no clear conclusion about the relationship between PD and essential tremor. In that case, our study was performed to evaluate the association of three SNPs that had previously been identified as genetic susceptibility factors for ET, in an eastern Chinese Han population of PD. However, we found that only SLC1A2 rs3794087 showed significant correlation with PD patients from East China and that this ET SNP appeared to decrease the risk of PD. There were no differences in the frequency of rs9652490 (*LINGO1*) and rs17590046 (*PPARGC1A*) between PD patients and healthy controls.

The leucine‐rich repeat and Ig domain containing 1 gene (*LINGO1*) was reported as the first genetic evidence of a link between ET and PD by Wszolek and colleagues.^17^ The SNP of rs9652490 is a part of the Nogo receptor complex, which is involved in inhibition of oligodendrocyte differentiation, axonal myelination and regeneration, and dopaminergic neuronal survival.^18^ Many studies have been performed to elucidate the role of *LINGO1* in ET and PD. Among them, it is worth noting that one study performed in Chinese patients found the G allele of rs9652490 has a protective effect on Parkinson's disease,^19^ whereas original studies found this allele to be risky for essential tremor.^8^ A meta‐analysis suggested a protective role for the rs9652490GG genotype in PD (HR = 0.70, *P* = .028).^20^ However, a study of the rs9652490 SNP in an Austrian sample of PD patients and healthy subjects failed to demonstrate a link between PD or the tremor‐based subgroup of PD.^18^ Given these conflicting results, the role of *LINGO1* in PD and ET needs to be further explored.

Solute carrier family 1‐glial affinity glutamate transporter‐member 2 (*SLC1A*2) encodes the major glutamate reuptake transporter EAAT2 of the brain, which removes glutamate from the synaptic cleft. It is notable that EAAT2 is strongly expressed in the inferior olive, which is implicated in rhythm generation in ET, but not in the substantia nigra. The SNP rs3794087 of *SLC1A2* was found to be associated with ET in a GWAS study from a European population, with an odds ratio (OR) of 1.4.^7^ However, results related to the role of *SLC1A2* rs3794087 in PD were inconsistent. Appenzeller et al^21^ were the first to suggest that there was no association between polymorphisms in *SLC1A2* and PD. In addition, Tan et al^22^ and Cheng et al^23^ also found a *SLC1A2* variant associated with essential tremor but not Parkinson's disease in a Chinese population. Whereas Xu et al^24^ suggested that *SLC1A2* rs3794087 might decrease the risk for PD in a northern Chinese cohort. Our result corroborates these latter findings. Genetic heterogeneity of ET and PD in various populations may explain, to a large extent, controversial results in these studies. Since our cohort is mainly from the eastern regions of China, additional studies involving populations from different ethnic origins and regions are needed to elucidate the association between rs3794087 of *SLC1A2* and PD.

Peroxisome proliferator‐activated receptor gamma coactivator 1‐alpha (PPARGC1A), or PGC‐1a, regulates mitochondrial biogenesis and suppresses oxidative stress.^25^ Accumulation of misfolded proteins has been shown to influence neuronal survival and vulnerability in PD.^26^ It is also reported that PGC‐1a plays a protective role against 1‐methyl‐4‐phenyl‐1,2,3,6‐tetrahydropyridine (MPTP) toxicity, which has been shown to be a causative factor of PD.^27^, ^28^ Rs17590046 in *PPARGC1A* was found to be significantly associated with increased risk of ET through a GWAS in an European population.^10^ Recently, a study in Singapore verified the role of rs17590046 in ET patients with Asian ancestry.^29^ Zhang et al^30^ were the first to show that rs17590046 was not associated with PD in a Chinese population. Later, Ross et al screened variants from 22 top SNPs identified in a ET GWAS in a cohort of French and French‐Canadian PD patients. They found that none of these variants including rs17590046 were significantly associated with PD.^31^ Our study also failed to identify a relationship between rs17590046 and PD.

## 5 CONCLUSION

In conclusion, our data show that the ET polymorphism, rs3794087 (SLC1A2), reduces the risk for PD in the Eastern Chinese population and that the other two polymorphisms do not show an association with PD. Due to the limited sample size and racial heterogeneity of our cohort, additional studies may help to further illuminate the relationship between PD and these variants.

## CONFLICTS OF INTEREST

The authors declare no conflicts of interest.
